# Effect of electroacupuncture stimulation at Zusanli acupoint (ST36) on gastric motility: possible through PKC and MAPK signal transduction pathways

**DOI:** 10.1186/1472-6882-14-137

**Published:** 2014-04-17

**Authors:** Qi Yang, Yan-Dong Xie, Ming-xin Zhang, Bo Huang, Chao Zhang, Hui-Yan Li, Rong Zhang, Ming Qin, Yu-Xin Huang, Jing-Jie Wang

**Affiliations:** 1 Department of Gastroenterology, Tangdu Hospital of the Forth Military Medical University, Xi’an, Shanxi 710038, China; 2 Department of Neurosurgery, Tangdu Hospital of the Forth Military Medical University, Xi’an, Shanxi 710038, China

**Keywords:** Electroacupuncture, Zusanli, Gastric motility, PKC, MAPK

## Abstract

**Background:**

Electroacupuncture (EA) stimulation has been shown to have a great therapeutic potential for treating gastrointestinal motility disorders. However, no evidence has clarified the mechanisms contributing to the effects of EA stimulation at the Zusanli acupoint (ST.36). This study was designed to investigate the regulative effect of EA stimulation at the ST.36 on gastric motility and to explore its possible mechanisms.

**Methods:**

Thirty Sprague-Dawley rats were randomly divided into three groups: the ST.36 group, the non-acupoint group, and the control group. EA stimulation was set at 2 Hz, continuous mode, and 1 V for 30 min. The frequency and average peak amplitude of gastric motility were measured by electrogastrography. The protein kinase C (PKC) and mitogen-activated protein kinase (MAPK) signaling pathways were assessed using real-time polymerase chain reactions. Caldesmon (CaD) and calponin (CaP) protein expression in the gastric antrum were detected on Western blots. A Computed Video Processing System was used to evaluate morphological changes in smooth muscle cells (SMCs) from the gastric antrum.

**Results:**

EA stimulation at ST.36 had a dual effect on the frequency and average peak amplitude. Additionally, EA stimulation at ST.36 regulated the expression of some genes in the PKC and MAPK signaling pathways, and it regulated the expression of the CaD and CaP proteins. EA serum induced SMC contractility. Promotion of gastric motility may correlate with up-regulation of MAPK6 (ERK3), MAPK13, and Prostaglandin-endoperoxide synthase 2 (PTGS2) gene expression, and the down-regulation of the collagen, type I, alpha 1 (COL1A1) gene and CaD and CaP protein expression. Inhibition of gastric motility may correlate with down-regulation of the Interleukin-1 receptor type 2 (IL1R2) and Matrix metalloproteinase-9 (MMP9) genes, and up-regulation of CaD and CaP protein expression.

**Conclusions:**

EA stimulation at ST.36 regulated gastric motility, and the effects were both promoting and inhibiting in rats. The possible mechanisms may correlate with the PKC and MAPK signal transduction pathways.

## Background

Gastric motility is one of the most critical physiological functions of the human gut. Without coordinated motility, the digestion and absorption of dietary nutrients cannot take place. The regulation of gastrointestinal motility is complicated and involves the contraction of smooth muscle cells (SMCs). The contraction of SMCs is primarily regulated by transient changes in the intracellular Ca^2+^ concentration [1]. There are two major pathways involved in this mechanism, namely, the RhoA-Rho kinase pathway and the protein kinase C (PKC) pathway [2]. Calponin (CaP), a well established *in vitro* substrate for signaling proteins by PKC [3], directly interacts with PKC [4]. Caldesmon (CaD) is an actin and myosin binding protein that exists in two isoforms, which are generated by alternative splicing [5]. There is accumulating evidence for a secondary pathway in the regulation of smooth muscle contraction that is PKC dependent, and this pathway may be mediated by CaP and CaD activation [6-9]. Mitogen-activated protein kinase (MAPK) signaling pathways have also been implicated in SMC contraction [10]. There are three major groups of distinctly regulated MAPKs that lead to altered gene expression. The extracellular signal related kinases 1 and 2 (ERK1/2), the C-jun terminal kinase (JNK), and the p38 MAPK are known to play important roles in the intracellular signaling response to extracellular stimuli [11]. In addition, CaP may facilitate ERK-dependent signaling, thus playing a significant role in the regulation of SMC contraction [12].

Acupuncture, which has been used for thousands of years in China, is increasingly used worldwide for the management of various diseases [13]. It is believed that stimulation of an acupoint can directly affect relevant organs and achieve the effect of acupuncture therapy. EA is a combined procedure that stimulates an acupoint with electrical stimulation instead of with manual manipulations of needles. Numerous studies have evaluated the effects and mechanisms of EA on gastric motility [14-20]. Based on the evidence from these studies, EA stimulation has been shown to have a great therapeutic potential for treating gastrointestinal motility disorders. The Zusanli (ST.36) is one of the most commonly used acupoints for gastrointestinal diseases. According to the theory of Traditional Chinese Medicine (TCM), there is a relationship between ST.36 and the function of the gastrointestinal tract. To date, however, no evidence has clarified the exact mechanisms contributing to the effects of EA stimulation.

Therefore, by examining morphologic changes and myoelectrical activity, the present study aimed to evaluate the regulative effect of EA stimulation at the ST.36 acupoint on gastric motility in rats and to explore its possible mechanisms.

## Methods

### Animals and reagents

Thirty adult male Sprague-Dawley (SD) rats weighing 180–220 g were maintained on a 12-h light-dark cycle at 25 ± 2°C and 60% humidity with free access to food and water. SD rats were purchased from the Experimental Animal Center of the Fourth Military Medical University. All animal experiments were carried out in accordance with the institutional guidelines of the Fourth Military Medical University for the care and use of laboratory animals. Approval of the study protocol was obtained from the Ethics Committee for Animal Research, Fourth Military Medical University, China. Animals were randomly allocated into three groups: the ST.36 group, the non-acupoint group, and the control group.

Collagenase II, trypsin inhibitor, dithiothreitol alcohol sugar, bovine serum albumin, calcium-free phosphate buffered saline (PBS), glutaraldehyde, and Trypan blue were purchased from Sigma Chemical Co. The Total RNA Extraction Kit, SuperScript III Reverse Transcriptase, and SuperArray PCR Master Mix were purchased from Invitrogen Co. Anti-goat, anti-mouse, and anti-rabbit antibodies were purchased from Shanghai Kangcheng Bio-Engineering Co.

### EA stimulation procedure

The ST.36 was located approximately 5 mm lateral to the fibula of the hind limbs, while the non-acupoint was located on the hind foot next to the ST.36 open 5 mm [21]. The needles were inserted 5 mm deep into the muscle layer of the selected acupoint and were stimulated by EA at 2 Hz and 2 V using an electrical stimulator (G6805-2A; Shanghai Huayi Medical Instrument Factory, Shanghai, China). Each stimulation lasted 20 min in continuous mode, and stimulation was carried out 1 time/day for 5 days. The rats in control group were submitted to immobilization alone without stimulation for 30 min at the same time each day.

### Electrogastrography

Electrogastrography was applied using the Multichannel Physiologic Signal Acquisition and Processing System (mode RM-6280) after the last stimulation on the fifth day. Following a 12-h fast (with free access to water) and 6 h without drink, the rats were anesthetized intraperitoneally (i.p.) with 10% urethane at a dose of 1 g/kg body and then immobilized. The first electrode was fixed to the tail, and guided electrodes were implanted on the serosal surface of the stomach 0.3 - 0.5 cm above the pylorus. The electrodes were covered with 37°C normal saline gauze, connected with wire. The experimental parameters used were as follows: frequency 400 Hz, filter 10 Hz, scanning speed 2 s/division, sensitivity 25 mV, time constant direct current, and oscilloscope recording time 1 h [18].

### Real-time polymerase chain reaction (PCR) analysis

After the last stimulation with EA, the rats were sacrificed. For control group and non-acupoint group, one rat was randomly selected respectively. For EA group, we first divided it into EA promoting group and EA inhibiting group according to the results of the electrogastrography. And then, there rats were randomly selected from both groups. Gastric antrum tissues (from the pyloric 0.8 - 1 cm) were cut into pieces that were approximately 0.3 cm × 0.5 cm in size and immediately placed in -70°C liquid nitrogen. Total RNA samples were extracted using TRIZOL from different groups according to the manufacturer’s instructions. RNA quality was assessed spectrophotometrically on the basis of the A260/A280 ratio. RNA samples were checked for integrity of the 18S and 28S RNA by gel electrophoresis. Total RNA (1.5 μg) was used to generate first-strand cDNA with SuperScript III Reverse Transcriptase (1 μl) from the isolated RNA. The real-time PCR reactions were prepared using a SuperArray PCR Master Mix (Cat. No. PA-112) containing the PKC and MAPK signal pathway genes. The diluted cDNA (102 μl) was added to each hole corresponding to the PCR chip. Then the PCR chips were placed into the real-time PCR instrument for the PCR reaction. The cycling conditions were as follows: 95°C for 10 min, 60°C for 15 s, and 60°C for 1 min (40 cycles). Differences in gene expression, expressed as fold-changes, were calculated using the 2^-ΔΔCt^ method.

### Western blots of CaD and CaP

The CaD and CaP protein levels were detected by Western blotting. After treatment with EA, the rats were sacrificed. The rats were then fixed, followed by incision of the abdominal skin and peritoneum. The viscera were exposed, and the central 1/3 of the gastric body was cut into small pieces that were approximately 0.3 cm × 0.5 cm in size. The pieces were immediately stored at -70°C in liquid nitrogen. Briefly, cell extracts (30 μg) from the upper 1/3 of the middle of the gastric body were separated by sodium dodecyl sulfate-polyacrylamide gel electrophoresis (SDS-PAGE) and then transferred to nitrocellulose membranes. The filters were blocked with Tris-buffered saline (TBS)/5% milk, followed by incubation with polyclonal antibodies against CaD (1:3000 dilution), CaP (1:3000 dilution), and β-actin (1:10000). After the membranes were washed, they were incubated with secondary peroxidase-conjugated antibodies diluted 1:5000 in TBS with 0.01% Tween 20. The antibody detection system was used, and the membranes were exposed to X-ray film according to the manufacturer’s instructions.

### Serum preparation

Serum was prepared as previously described with modification [22]. After the last stimulation with EA, all rats were sacrificed. Next, 6 ml of blood was taken from the carotid of each rat. The blood was kept still for 1 h, and then centrifuged at 3000 rpm for 15 min. The serum was collected, filtered to remove bacteria, and 1.5 ml was packed in an EP tube, which was stored at -70°C in liquid nitrogen.

### Effects of EA serum on SMC contractility

Five normal SD rats were anesthetized i.p. with 10% urethane at a dose of 1 g/kg body weight. After being anesthetized, the abdominal skin and peritoneum were incised, and the viscera were exposed. The whole stomach was cut out, and the gastric antrum tissues were isolated and immediately placed in oxygen saturated PBS with penicillin (100 U/ml) and streptomycin (100 μg/ml) at 4°C. After being rinsed, the tissues were transferred to oxygen saturated HEPES, spread and fixed on a silica gel plate with a fine-needle. The mucosal layer was carefully cut off, exposing the circular mucosa, which was clipped into 9 - 10 muscle strips (1 mm × 4 mm), placed in Tyrode’s solution, and kept at 4°C in the refrigerator for approximately 15 min. The muscle strips were digested in 1% collagenase type II, 0.05% trypsin inhibitor, 0.05% dithiothreitol alcohol sugar, and 0.2% bovine serum albumin enzyme in a water bath for 25 min at 36°C. After digestion, the mixture was rinsed with enzyme-free HEPES, incubated for 30 min, and filtered with a 200 screen mesh to collect the free single gastric SMCs. Cell viability was measured with the Trypan blue test. The living cells were more than 95% at a density of 10^6^ cells/ml.

The isolated SMCs were randomly divided into the normal serum group, ST.36 serum group, and non-acupoint serum group. SMCs suspensions (50 μl) at a density of 1.0 × 10^6^ cells/ml were placed in 96-well plates. Each serum sample was inactivated after 30 min in a 56°C water bath, and then diluted 1:2 with HEPES. Serum (50 μl) from the different groups was added to SMCs, mixed with a pipette for 30 s, and fixed with 30 μl 2.5% glutaraldehyde. Cell length was assessed with a Computed Video Processing System (DP2-BSW), which was used to calculate the shrinkage percentage. The cell shrinkage percentage = (cell length before treatment - cell length after treatment)/cell length before treatment × 100%.

### Statistical analysis

Data were expressed as mean ± S.D. One-way analysis of variance followed by Bonferroni’s multiple comparison test was applied with SPSS 10.0 software. The ΔCt values obtained from the real-time PCR, which were used to calculate the fold-change differences (expressed here as percent of control), were also used for the statistical analysis. A *p*-value < 0.05 or a fold-change > 2 was defined as statistically significant for all analyses.

## Results

### Effects of EA on gastric motility

EA at ST.36 produced significant changes in the absolute value of the average peak amplitude and frequency compared with the control or non-acupoint groups (*P* < 0.01). In contrast, EA at the non-acupoint did not induce significant changes in gastric motility compared with the control group (Additional file 1: Figure S1). Two types of gastric motor patterns were observed for the ST.36 group in the 10 rats tested according the average peak: rats with positive average peak were judged to promoting pattern, while rats with negative average peak were judged to promoting pattern. The results showed 5 (50.0%) rats showed the ST.36 promoting pattern and 5 (50.0%) rats showed the ST.36 inhibiting pattern. In rats with the ST.36 promoting pattern, the average peak amplitude and frequency values were 47.13 ± 0.44 and 0.19 ± 0.06, respectively. In rats with the ST.36 inhibiting pattern, the average peak amplitude and frequency values were -43.41 ± 0.62 and 0.17 ± 0.03, respectively (Additional file 2: Table S1).

### Effects of EA on the PKC and MAPK signaling pathways

EA at ST.36 significantly regulated the expression of some genes in the PKC and MAPK signaling pathways. Table 1 lists the results from the microarray analysis of the ST.36 promoting group (as compared to the control group). The expression of many genes was increased after EA stimulation, including ERK3, MAPK13, IL1R1, PRKCA, and PTGS2. Other genes showed decreased expression after EA stimulation, including IL1R2. Table 2 lists the results from the microarray analysis of the ST.36 inhibiting group (as compared to the control group). The gene expression of IL1R2 and MMP9 were decreased after EA stimulation.

**Table 1:** Gene change comparing EA promoting group with control group

**Signal pathway**	**Gene**	**Sites**	**EA/control (Fold change)**	**EA/control (Fold up- or down-regulation)**
MAPK	MAPK6 (ERK3)	D02	2.39	2.39
MAPK	MAPK13	D10	2.86	2.86
PKC	IL1R1	F08	2.18	2.18
PKC	PRKCA	G01	2.40	2.40
PKC	PTGS2	G02	2.72	2.72
PKC	IL1R2	F09	0.26	-3.85

Fold-change (2^-ΔΔCt^) is the normalized gene expression (2^-ΔCt^) in the test sample divided by the normalized gene expression (2^-ΔCt^) in the control sample. Fold-regulation represents the fold-change results in a biologically meaningful way. Fold-change values greater than one indicate a positive- or up-regulation, and the fold-regulation is equal to the fold-change. Fold-change values less than one indicate a negative or down-regulation, and the fold-regulation is the negative inverse of the fold-change.

**Table 2:** Gene change comparing EA inhibitory group with control group

**Signal pathway**	**Gene**	**Sites**	**EA/control (Fold change)**	**EA/control (Fold up- or down-regulation)**
PKC	IL1R2	F09	0.24	-4.26
PKC	MMP9	F11	0.21	-4.68

Fold-change (2^-ΔΔCt^) is the normalized gene expression (2^-ΔCt^) in the test sample divided by the normalized gene expression (2^-ΔCt^) in the control sample. Fold-regulation represents the fold-change results in a biologically meaningful way. Fold-change values greater than one indicate a positive- or up-regulation, and the fold-regulation is equal to the fold-change. Fold-change values less than one indicate a negative or down-regulation, and the fold-regulation is the negative inverse of the fold-change.

Table 3 lists the results from the microarray analysis of the ST.36 promoting group (as compared to the non-acupoint group). The expression of the COL1A1 gene was increased after EA stimulation. The expression of the IL1R2 and SERPINE1 genes were decreased after EA stimulation. Table 4 lists results from the microarray analysis of the ST.36 inhibiting group (as compared to the non-acupoint group). Many of the genes showed decreased expression after EA stimulation, including the ERK3, JNK3, p38bMAPK, MAPK13, p38MAPK, ERK1, FGF2, IL1R1, IL1R2, MYC, PRKCA, and PTGS2 genes. The expression of the COL1A1 gene was increased after EA stimulation.

**Table 3:** Gene change comparing EA promoting group with non-acupoint group

**Signal pathway**	**Gene**	**Sites**	**EA/Non-acupoint (Fold change)**	**EA/Non-acupoint (Fold up- or down-regulation)**
PKC	COL1A1	F05	3.60	3.60
PKC	IL1R2	F09	0.39	-2.54
PKC	SERPINE1	G04	0.48	-2.07

Fold-change (2^-ΔΔCt^) is the normalized gene expression (2^-ΔCt^) in the test sample divided by the normalized gene expression (2^-ΔCt^) in the control sample. Fold-regulation represents the fold-change results in a biologically meaningful way. Fold-change values greater than one indicate a positive- or up-regulation, and the fold-regulation is equal to the fold-change. Fold-change values less than one indicate a negative or down-regulation, and the fold-regulation is the negative inverse of the fold-change.

**Table 4:** Gene change comparing EA inhibitory group with non-acupoint group

**Signal pathway**	**Gene**	**Sites**	**EA/Non-acupoint (Fold change)**	**EA/Non-acupoint (Fold up- or down-regulation)**
MAPK	MAPK6 (ERK3)	D02	0.18	-5.67
MAPK	MAPK10 (JNK3)	D07	0.42	-2.40
MAPK	MAPK11 (p38bMAPK)	D08	0.39	-2.58
MAPK	MAPK13	D10	0.28	-3.58
MAPK	MAPK3 (ERK1)	G11	0.26	-3.83
MAPK	MAPK14 (p38MAPK)	D11	0.47	-2.13
PKC	FGF2	F07	0.37	-2.67
PKC	IL1R1	F08	0.30	-3.31
PKC	IL1R2	F09	0.36	-2.81
PKC	MMP9	F11	0.22	-4.48
PKC	MYC	F12	0.39	-2.59
PKC	PRKCA	G01	0.23	-4.27
PKC	PTGS2	G02	0.19	-5.36
PKC	COL1A1	F05	4.81	4.81

Fold-change (2^-ΔΔCt^) is the normalized gene expression (2^-ΔCt^) in the test sample divided by the normalized gene expression (2^-ΔCt^) in the control sample. Fold-regulation represents the fold-change results in a biologically meaningful way. Fold-change values greater than one indicate a positive- or up-regulation, and the fold-regulation is equal to the fold-change. Fold-change values less than one indicate a negative or down-regulation, and the fold-regulation is the negative inverse of the fold-change.

### Effects of EA on CaD and CaP protein expression

**Figure 1:**
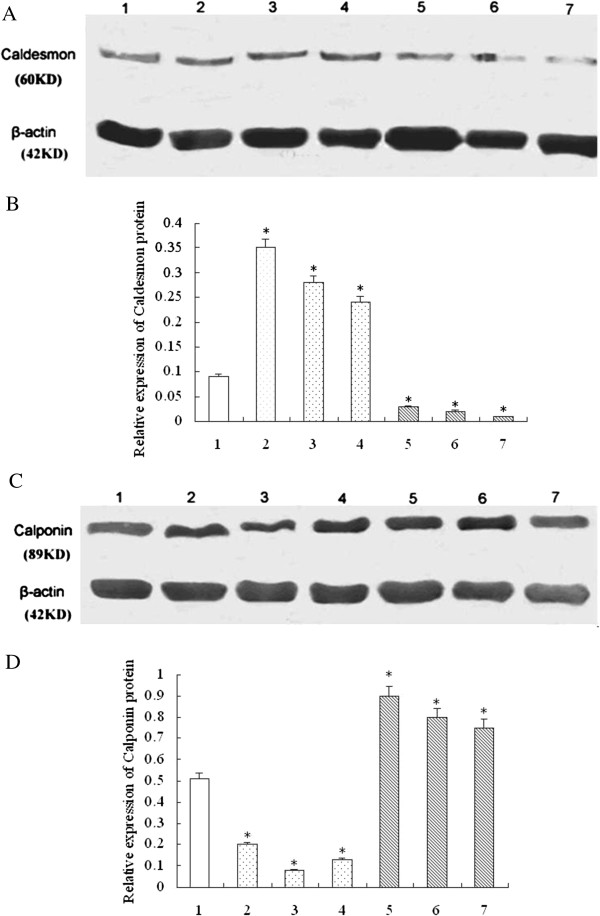
**Effects of EA stimulation on Caldesmon (CaD) and Calponin (CaP) protein levels. A**. Expression of Caldesmon protein levels by Western blotting. **B**. Densitometric analysis Caldesmon protein levels normalized by β-actin; **C**. Expression of Calponin protein levels by Western blotting; **D** Densitometric analysis Calponin protein levels normalized by β-actin. CaD: Caldesmon; CaP: calponin; 1: non-acupoint group; 2–4: EA inhibiting group; 5–7: EA promoting group. *P < 0.05 compard to non-acupoint group.

Stimulation with EA significantly increased CaD and CaP protein expression in the ST.36 inhibiting group compared with the non-acupoint group, whereas stimulation with EA significantly decreased CaD and CaP protein expression in ST.36 promoting group compared with the non-acupoint group (Figure 1A, B, C, D).

### Effects of EA serum on SMC contractility

As shown in Additional file 3: Figure S2 and Additional file 4: Table S2, cell morphology was observed under the light microscope after treatment with different types of serum. There were no differences in cell length before the addition of serum (p > 0.05). ST.36 serum increased SMC contractility (60.22 ± 3.84 μm) compared with the control serum (96.02 ± 6.24 μm) or the non-acupoint serum (97.33 ± 6.97 μm). The contraction percentage was 41% after stimulation with ST.36 serum, whereas the contraction percentages were 7% and 10% after stimulation with either the non-acupoint serum or control serum, respectively.

## Discussion

The present study demonstrated that EA stimulation at ST.36 had a regulative effect on gastric motility in rats that was either stimulatory or inhibitory. The stimulating effect of EA may act through the PKC and MAPK signaling pathways.

The effects of EA on gastric motility have been extensively studied in both animals and humans due to the availability of electrogastrography. It is well known that gastric myoelectrical activity consists of slow waves and spikes. Spikes are directly associated with the appearance of contractions. Slow waves do not evoke gastrointestinal contractility, but they determine the maximum frequency of gastric contractions.

The most often used acupoint in treating gastrointestinal disorders is the ST.36. It is commonly believed that acupuncture may exert promoting and inhibiting effects on gastrointestinal motility depending on the stimulated acupoint. In humans, it has been shown that EA stimulation at ST.36 had dual effects on gastric peristalsis that are either facilitative or inhibitory [15,23]. Although patients suffering from gastrointestinal diseases have benefited from the application of acupuncture at ST.36, but the exact molecular mechanisms are still unclear. In dogs, EA at ST.36 increased the regularity of gastric slow waves [24,25]. In conscious rats, EA stimulation at ST.36 induced dual effects, which were either facilitative or inhibitory, on gastric motility [26,27]. Further study revealed that the stimulatory effect is mediated via cholinergic pathways, while the inhibitory effect is independent of the sympathetic pathway [28]. The general theory of acupuncture medicine is premised on the concept of Yin and Yang, and acupuncture is believed to modify the imbalance between Yin and Yang. The effects of acupuncture on balancing Yin and Yang may be mediated via the interaction between the neurotransmission of GABA and glutamate in the brain stem.

In the current paper, EA stimulation at ST.36 was effective in regulating gastric motility, as demonstrated by the significant increase in peak amplitude and frequency. In addition, the lack of such a response when the same stimulation was performed at the non-acupoint was indicative of the specificity of this effect. According to the peak amplitude and frequency, the ST.36 group was further divided into promoting and inhibitory patterns, since EA showed a dual effect. To further explore the mechanisms by which EA regulates gastric motility, we isolated SMCs from the stomach and observed the effect of ST.36 serum on SMC contractility using serologic pharmacological testing methods. The results showed that ST.36 serum significantly increased SMC contractility; whereas the effects produced by normal or non-acupoint serum were not obvious.

Recent investigations suggested that the effects of EA stimulation are mediated through the opioid system [29] or through its stimulating effect on vagal activity [30]; however, the precise mechanisms by which EA affects gastric motility were not fully understood. Previous findings also indicated that PKC levels were increased after stimulation with EA [31]. Therefore, we selected the PKC and MAPK pathways as targets for mediating the stimulatory effects of EA on gastric mobility. Moreover, CaP and CaD may play a role in the regulation of gastrointestinal motility during physiological and pathological adaptation. Up-regulation of CaP and CaD expression inhibited gastrointestinal motility, and down-regulation of CaP and CaD expression promoted gastrointestinal motility [32,33]. ERK1/2, a member of the MAPK family, has been shown to play a significant role in the regulation of smooth muscle contraction [34]. According to our microarray results, promotion of gastric motility may correlate with up-regulation of MAPK6 (ERK3), MAPK13, and PTGS2 gene expression, and down-regulation of COL1A1 gene expression. Inhibition of gastric motility may correlate with down-regulation of IL1R2 and MMP9 gene expression. Western blots showed up-regulation of CaD and CaP protein expression inhibited gastric motility, whereas down-regulation of CaD and CaP protein expression promoted gastric motility. These results demonstrate that CaD and CaP may play a role in the regulation of gastrointestinal motility during physiological and pathological adaptation.

There were some limitations in the present study that should be mentioned. First, we did not investigate whether the inhibitors of PKC and MAPK could affect or block the regulatory effect of EA stimulation at ST.36 on gastric mobility. Further studies will be required to investigate the influences of PKC and MAPK inhibitors. Second, future investigations will focus on fully understanding the roles of each change in gene expression upon the regulation of gastric mobility.

## Conclusions

In summary, our findings demonstrate that EA stimulation at ST.36 regulated gastric motility. EA stimulation at ST.36 exerted dual effects on gastric motility that were either stimulatory or inhibitory. In addition, regulation of gastric motility may correlate with the PKC and MAPK signal transduction pathways.

## Competing interests

The authors declare that they have no competing interests.

## Authors’ contributions

QY carried out the molecular genetic studies, participated in the sequence alignment and drafted the manuscript. YDX, MXZ, BH carried out the immunoassays. CZ, HYLi, RZ participated in the sequence alignment. MQ, YXH participated in the design of the study and performed the statistical analysis. JJW conceived of the study, and participated in its design and coordination and helped to draft the manuscript. All authors read and approved the final manuscript.

## Pre-publication history

The pre-publication history for this paper can be accessed here:

http://www.biomedcentral.com/1472-6882/14/137/prepub

## Supplementary Material

Additional file 1: Figure S1 Effects of EA stimulation on gastric motility. Gastric motility was measured by electrogastrography. A. Control, B. ST.36, C. Non-acupoint. Stimulation with EA at ST.36 increased the average peak amplitude and frequency. Gastric waves showed dual changes in the ST.36 group compared with the control or non-acupoint group. Values are expressed as the mean ± S.D. (*n* = 10). *p < 0.05, **p < 0.01 *vs*. control group. ^#^*p* < 0.05, ^##^*p* < 0.01 *vs.* non-acupoint group. Click here for file

Additional file 2: Table S1 Effects of EA on gastric myoelectrical activity. The ST.36 group was further divided into EA promoting and EA inhibiting groups according to the gastric waves. Values are expressed as mean ± S.D (*n* = 5). Click here for file

Additional file 3: Figure S2 Effects of EA stimulation on gastric SMC contractility. Cell morphology was observed using a light microscope (×200 magnification). The length of the cell and the contraction percentage was measured with a Computer Image Analysis System. O. SMCs; A. SMCs + control serum; B. SMCs + ST.36 serum; C. SMCs + non-acupoint serum. Click here for file

Additional file 4: Table S2 Effects of serum on SMC contractility. As assessed by the Computed Video Processing System, Zusanli serum resulted in significant contractility in SMCs. Results are expressed as the mean ± S.D (*n* = 10). *p < 0.05 **p < 0.01 *vs.* control serum group. ^#^*p* < 0.05, ^##^*p* < 0.01 *vs.* non-acupoint serum. Click here for file
