# Supplementary figures and images for: Effect of electroacupuncture stimulation at Zusanli acupoint (ST36) on gastric motility: possible through PKC and MAPK signal transduction pathways
Source: BMC Complement Altern Med. 2014 Apr 17;14:137. doi: 10.1186/1472-6882-14-137 (PMC4021071; doi:10.1186/1472-6882-14-137)

**Figure S1**

**
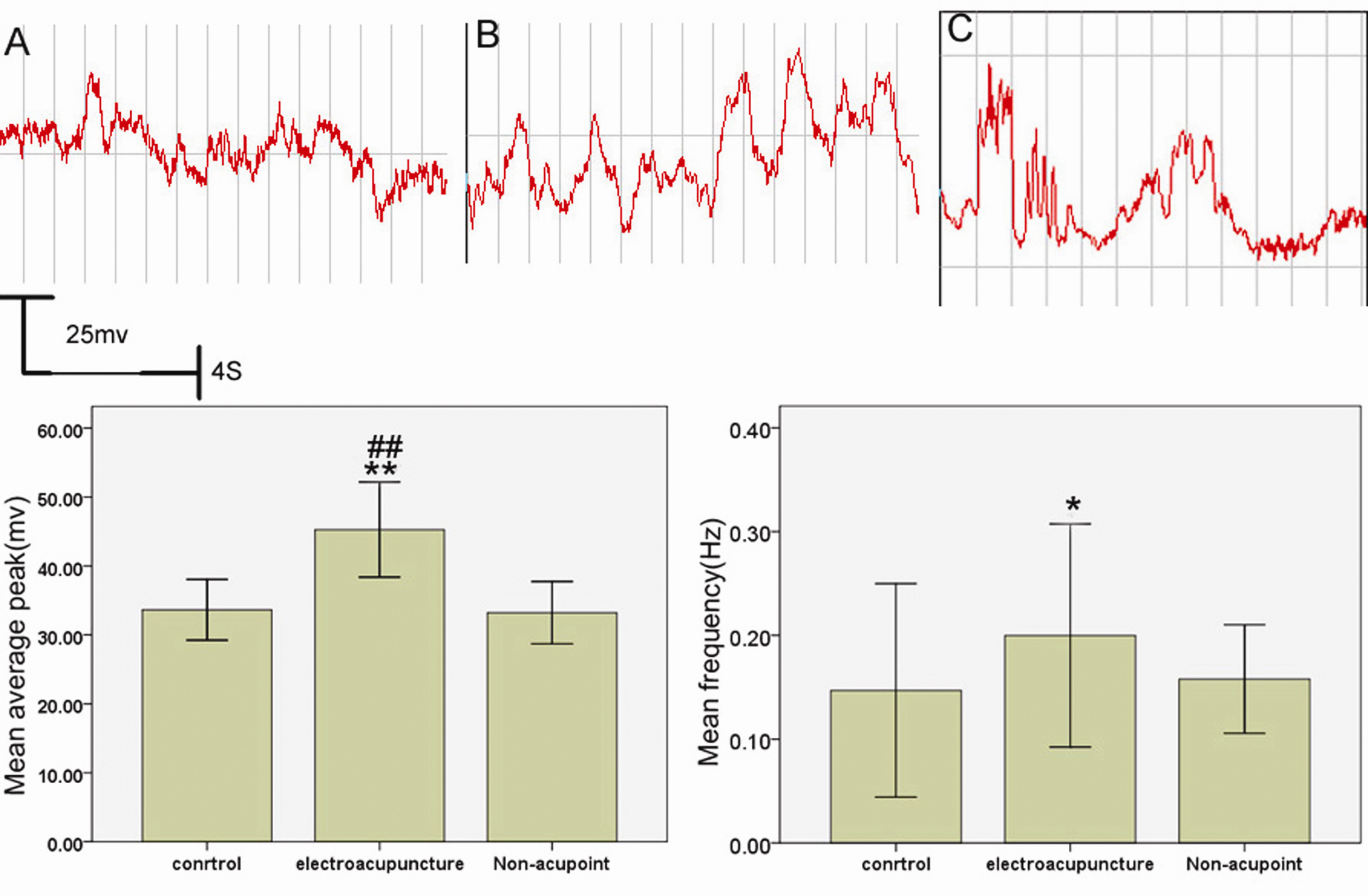
**

Supplement: Additional file 1: Figure S1 — Effects of EA stimulation on gastric motility. Gastric motility was measured by electrogastrography. A. Control, B. ST.36, C. Non-acupoint. Stimulation with EA at ST.36 increased the average peak amplitude and frequency. Gastric waves showed dual changes in the ST.36 group compared with the control or non-acupoint group. Values are expressed as the mean ± S.D. (n = 10). *p < 0.05, **p < 0.01 vs. control group. #p < 0.05, ##p < 0.01 vs. non-acupoint group. [file 1472-6882-14-137-S1.doc]

**Figure S2**

**
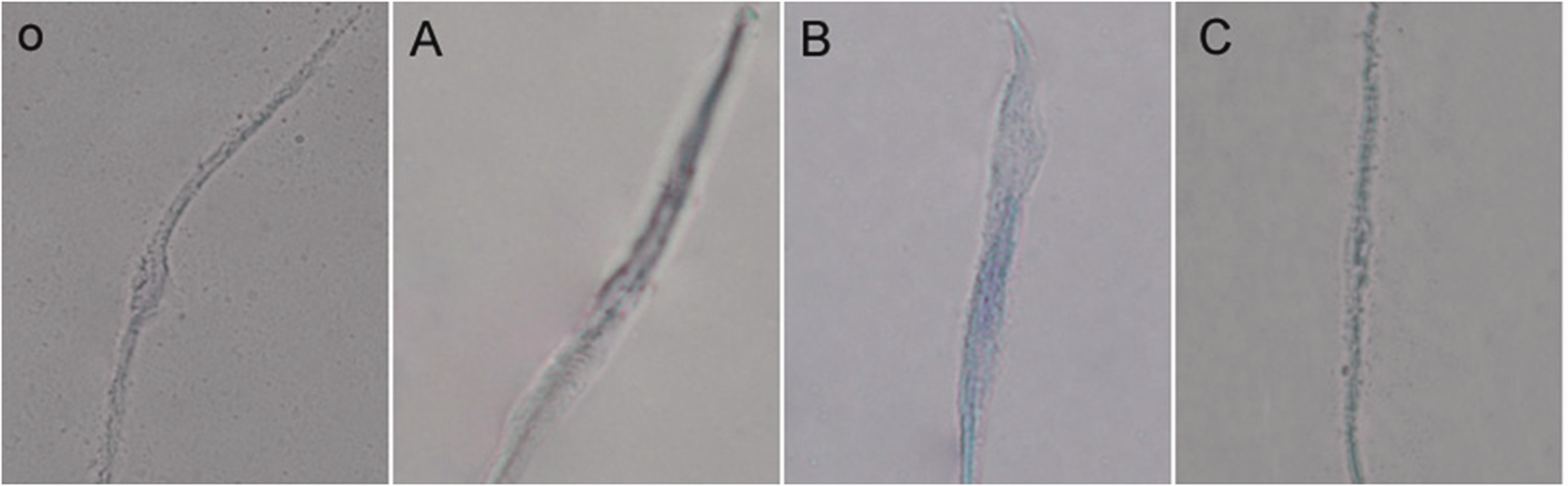
**

Supplement: Additional file 3: Figure S2 — Effects of EA stimulation on gastric SMC contractility. Cell morphology was observed using a light microscope (×200 magnification). The length of the cell and the contraction percentage was measured with a Computer Image Analysis System. O. SMCs; A. SMCs + control serum; B. SMCs + ST.36 serum; C. SMCs + non-acupoint serum. [file 1472-6882-14-137-S3.doc]
