# Supplementary material for: Effect of electroacupuncture stimulation at Zusanli acupoint (ST36) on gastric motility: possible through PKC and MAPK signal transduction pathways
Source: BMC Complement Altern Med. 2014 Apr 17;14:137. doi: 10.1186/1472-6882-14-137 (PMC4021071; doi:10.1186/1472-6882-14-137)
Supplement: Additional file 2: Table S1 — Effects of EA on gastric myoelectrical activity. The ST.36 group was further divided into EA promoting and EA inhibiting groups according to the gastric waves. Values are expressed as mean ± S.D (n = 5). [file 1472-6882-14-137-S2.doc]

**Table S1. Effects of EA on gastric myoelectrical activity**

| Group | Average peak amplitude (Mv) | Average frequency (Hz) |
| --- | --- | --- |
| Promoting | 45.93 ± 3.69 | 0.24 ± 0.04 |
| Inhibiting | -44.61 ± 3.49 | 0.17 ± 0.04 |
