# Supplementary material for: Effect of electroacupuncture stimulation at Zusanli acupoint (ST36) on gastric motility: possible through PKC and MAPK signal transduction pathways
Source: BMC Complement Altern Med. 2014 Apr 17;14:137. doi: 10.1186/1472-6882-14-137 (PMC4021071; doi:10.1186/1472-6882-14-137)
Supplement: Additional file 4: Table S2 — Effects of serum on SMC contractility. As assessed by the Computed Video Processing System, Zusanli serum resulted in significant contractility in SMCs. Results are expressed as the mean ± S.D (n = 10). *p < 0.05 **p < 0.01 vs. control serum group. #p < 0.05, ##p < 0.01 vs. non-acupoint serum. [file 1472-6882-14-137-S4.doc]

**Table S2. Effects of serum on SMC** contractility

| Group | Cell length before adding serum (μm) | Cell length after adding serum (μm) | Contraction percentage (%) |
| --- | --- | --- | --- |
| Control serum | 106.15 ± 6.16 | 96.02 ± 6.24 ^##^ | 10 |
| Zusanli serum | 102.05 ± 3.91 | 60.22 ± 3.84** ^##^ | 41 |
| Non-acupoint serum | 104.33 ± 6.18 | 97.33 ± 6.97 | 7 |
